# Effect of Common Genetic Variants of Growth Arrest-Specific 6 Gene on Insulin Resistance, Obesity and Type 2 Diabetes in an Asian Population

**DOI:** 10.1371/journal.pone.0135681

**Published:** 2015-08-18

**Authors:** Chang-Hsun Hsieh, Ren-Hua Chung, Wen-Jane Lee, Ming-Wei Lin, Lee-Ming Chuang, Thomas Quertermous, Themistocles Assimes, Yi-Jen Hung, Ya-Wen Yu

**Affiliations:** 1 Division of Endocrinology and Metabolism, Department of Internal Medicine, Tri-Service General Hospital, National Defense Medical Center, Taipei, Taiwan; 2 Division of Biostatistics and Bioinformatics, Institute of Population Health Sciences National Health Research Institutes, Miaoli Country, Taiwan; 3 Department of Medical Education and Research, Taichung Veterans General Hospital, Taichung, Taiwan; 4 Institute of Public Health, National Yang-Ming University, Taipei, Taiwan, and Department of Medical Research and Education, Taipei Veterans General Hospital, Taipei, Taiwan; 5 Department of Internal Medicine, National Taiwan University Hospital, Taipei, Taiwan, and Graduate Institute of Preventive Medicine, National Taiwan University School of Public Health, Taipei, Taiwan; 6 Department of Medicine, Division of Cardiovascular Medicine, Stanford University School of Medicine, Stanford, California, United States of America; 7 Stanford Cardiovascular Institute, Stanford University, Stanford, California, United States of America; Harvard Medical School, UNITED STATES

## Abstract

**Objectives:**

Growth arrest-specific 6 (Gas6), a vitamin K-dependent protein, has been implicated in systemic inflammation, obesity, and insulin resistance (IR). Data from recent studies suggest that polymorphisms in the *Gas6* gene are associated with cardiovascular disorders and type 2 diabetes (T2D). However, the association of *Gas6* gene variants with obesity, IR, and T2D development has not been explored.

**Materials and Methods:**

Four common single nucleotide polymorphisms (SNPs) in the *Gas6* gene were genotyped in 984 participants from the Stanford Asia-Pacific Program for Hypertension and Insulin Resistance (SAPPHIRe) family cohort. An insulin suppression test was performed to determine IR based on steady-state plasma glucose (SSPG). Associations between IR indices and obesity, and SNP genotypes, based on previously-reported data for this cohort (Phase I), were analyzed. In the present follow-up study (Phase II), the effects of gene variants of *Gas6* on the progression to T2D were explored in individuals who were free of T2D in Phase I. The mean follow-up period for Phase II was 5.7 years.

**Results:**

The mean age of the study population in Phase I was 49.5 years and 16.7% of individuals developed T2D during follow-up. After adjusting for covariates, three SNPs (rs8191973, rs8197974, and rs7323932) were found to be associated with SSPG levels (*p* = 0.007, *p* = 0.03, and *p* = 0.011, respectively). This association remained significant after multiple testing and showed a significant interaction with physical activity for SNP rs8191973. However, no other significant correlations were observed between *Gas6* polymorphisms and other indices of IR or obesity. A specific haplotype, AACG (from rs8191974, rs7323932, rs7331124, and rs8191973), was positively associated with SSPG levels (*p* = 0.0098). None of the polymorphisms were associated with an increased risk of T2D development.

**Conclusions:**

Our results suggest that *Gas6* gene variants are associated with IR, although their effects on subsequent progression to T2D were minimal in this prospective Asian cohort.

## Introduction

Growth arrest-specific 6 (Gas6) is a vitamin K-dependent plasma protein which plays an essential role in hemostasis [1]. Gas6 has been shown to be involved in regulating multiple cellular functions through binding to the Tyro-3, Axl, and Mer (TAM) family of receptor tyrosine kinases [2–4]. Moreover, Gas6 may also play an important role in atherosclerosis and the thrombotic process [5], [6]. Changes in plasma Gas6 levels have been associated with clinical disorders characterized by pro-inflammatory and pro-thrombotic events [7–9].

Recent studies in animal models have shown that Gas6/TAM signaling plays an important role in pathophysiological mechanisms underlying obesity-related inflammation and insulin resistance (IR) [10]. We previously demonstrated that plasma Gas6 levels are associated with glucose intolerance, markers of inflammation, and endothelial dysfunction (both in adults and adolescents) [11], [12]. These findings support a potential role for Gas6 in the pathogenesis of obesity, IR, and related complications.

The *Gas6* gene, which is localized to chromosome 13q34, was characterized more than two decades ago [1]. Previous studies have explored the associations between genetic variants of *Gas6* and various diseases states, including cardiovascular disorders [7], [13], [14]. Munoz and colleagues identified several single nucleotide polymorphisms (SNPs) of the *Gas6* gene, and found an association between SNP c.843 + 7G > A in intron 8 (rs8191974) and stroke [13]. Moreover, the possible protective roles of specific haplotypes of the *Gas6* gene against stroke have been reported [14]. Jiang and colleagues also provided evidence that the AA genotype of rs8191974 confers a lower risk for the development of acute coronary syndrome [7]. In addition, a recent study by Lee and colleagues explored the association between genetic variants of *Gas6* and glucose homeostasis in 278 adults with various levels of glucose intolerance, and they confirmed that individuals with the c.843 + 7AA genotype had a lower risk of developing type 2 diabetes (T2D) [15].

Both obesity and IR are independent risk factors for cardiovascular disorders and T2D. To date, in-depth studies on the role of the *Gas6* gene in obesity and IR are lacking. Thus, the aim of the present study was to explore the effects of genetic variations of *Gas6* on obesity and the risk of developing IR in an Asian population. The associations between *Gas6* polymorphisms and the propensity for developing T2D were also explored in subjects without diabetes.

## Materials and Methods

### Ethics statement

This study was approved by the Institutional Review Board at each participating site. Informed consent was obtained from all participants before enrollment, and all procedures and clinical investigations were conducted in accordance with the principles outlined in the Declaration of Helsinki.

### SAPPHIRe study cohort

The Stanford Asia-Pacific Program for Hypertension and Insulin Resistance (SAPPHIRe) was designed to investigate genetic determinants of hypertension and IR in Chinese and Japanese populations. The program enrolled over 1,300 sibling pairs that were either concordant (both with hypertension) or discordant (only one with hypertension) from Stanford (San Francisco Bay Area), Hawaii (the island of Oahu and outer islands), and Taiwan. The participants were classed as hypertensive if they had a systolic blood pressure ≥140 mmHg, or diastolic blood pressure ≥90 mmHg, or if they were taking medications for high blood pressure. In brief, probands with an age at the onset of hypertension between 35 and 60 years (including participants aged >60 years if medical records indicated the onset of hypertension at an age <60 years) were recruited. The exclusion criteria included pre-existing malignancies or major chronic systemic diseases (including T2D and chronic liver, renal, or heart disease). Detailed descriptions of the study cohort have been published previously [16]. After Phase I (which refers to the original study cohort), the Taiwanese study cohort were recalled for follow-up (Phase II). Approximately 750 participants were successfully recalled. Among the reasons for participants being lost to follow-up were death, difficulty in re-establishing contact, or refusal to continue in the study [17].

### Clinical measurements

Blood pressure was measured using a mercury sphygmomanometer three times, and the average of the second and third measurements was used. Demographic data including age (at assessment), gender, level of education, ethnicity, and lifestyle factors (including cigarette smoking, alcohol intake, and physical inactivity) were obtained by questionnaires as described previously [18]. Obesity was defined as a body mass index (BMI) (body weight [in kg] divided by square of body height [m]) ≥30). In addition to BMI, waist circumference and waist-hip ratio were also assessed for each participant (as these may be better indicators of central obesity which is more prevalent in Asian populations). Plasma glucose concentrations were determined by the glucose oxidase method on a Beckman Glucose Analyzer II (Beckman Instruments, Fullerton, CA, USA). The intra- and inter-assay coefficients of variation for glucose were 0.6% and 1.5%, respectively. Plasma insulin was measured using a commercial immunoradiometric kit (BioSource Europe, Nivelles, Belgium), and the intra- and inter-assay coefficients of variation for insulin were 2.2 and 6.5%, respectively. For all biochemical parameters, duplicate measurements were taken and average values obtained.

### Measurement of insulin resistance

The modified insulin suppression test (IST) was performed to measure IR as described by Shen and Reaven [19]. Briefly, the participants received a 180-minute infusion of somatostatin at a rate of 250 μg/h, and insulin (25 mU/m^2^/min) and glucose (240 mg/m^2^/min) were administered simultaneously (after an overnight fasting period). During the infusion, blood was initially sampled every 30 minutes and then at 10-minute intervals (from 150 to 180 minutes). These samples were used to determine the steady-state plasma glucose (SSPG) concentrations for each participant (with increasing SSPG values indicating increasing IR). Oral glucose tolerance tests (OGTTs) were performed on a yearly basis for each participant during the follow-up period. T2D was diagnosed as a fasting plasma glucose (FPG) ≥126 mg/dl and/or OGTT (2-hour post-challenge) levels ≥200 mg/dl, or if the participant was taking oral hypoglycemic agents to control diabetes. IR was also evaluated based on the homeostasis model assessment (HOMA), calculated as HOMA insulin resistance (HOMA-IR) = fasting plasma insulin (FPI) (ìU/ml) × FPG (mmol/l)/22.5 [20].

### Selection of SNPs and genotyping

To identify common tag SNPs, we selected SNPs from the HapMap CHB (Chinese Beijing) database (http://snpinfo.niehs.nih.gov/snpinfo/snptag.htm) with a minor allele frequency threshold of 0.1 and *r* ^*2*^ of 0.8. Two tag SNPs, rs7323932 and rs8191973, were selected; in addition, rs8191974 and rs7331124 were also selected for validation based on previous studies [7, 13–15]. The TaqMan SNP Genotyping Assay (Applied Biosystems) was used for SNP genotyping of the genes selected in this study. The assays were either pre-designed (obtained from Applied Biosystems) or custom-made (Assay-by-Design, ABI). Samples were assayed along with no-template control samples, and run on an AB 7900HT Fast Real-Time PCR System (Applied Biosystems) using the following conditions: 10 minutes at 95°C (enzyme activation) followed by 40 cycles at 92°C for 15 seconds and 60°C for 1 minute (annealing/extension). The allelic discrimination results were determined after amplification by performing an endpoint read.

### Statistical analysis

All data are expressed as mean ± standard deviation (SD) unless otherwise specified. Tests for Hardy-Weinberg equilibrium (HWE) were performed before marker-trait association analysis. One individual was randomly selected from each family, and the resulting 443 unrelated individuals were used for the HWE tests. PLINK [21], a comprehensive toolset for genome association analysis, was used to perform exact tests for HWE based on the 443 individuals. Trait value distributions that were highly skewed from normality (including FPI and HOMA-IR) were log-transformed before analysis. We used Pedigree Based Association Testing (PBAT) to perform family-based association tests for single-marker and haplotype analyses [22]. PBAT compares the trait and SNP correlation in siblings to the expected value, conditional on the parental genotypes. Age, gender, site, and ethnic population were used as covariates for the association tests of SNPs with obesity, while age, gender, BMI, site and ethnicity were used as covariates for the association tests of SNPs with SSPG, FPI, and HOMA-IR. When covariates are used in PBAT, trait as the response and covariates as explanatory variables are used to fit the regression model; the residuals are then used as the trait for PBAT. Four marker haplotypes were analyzed, and age, gender, BMI, site, and ethnicity were used as covariates. Hypertensive status was included as an extra covariate. An additive model was assumed for the single-marker analysis. To estimate the odds ratios of the SNPs for obesity, we used generalized estimating equations (GEEs). To account for the correlation structures in families, obesity was used as the binary outcome and SNPs and covariates were used as explanatory variables in the regression model. Adjusted means for the continuous traits (i.e. SSPG, FPI and HOMA-IR) within each genotype category, and the standard errors for the adjusted means were also calculated based on the results from the GEE models (trait was used as the continuous outcome, and SNPs and covariates were used as explanatory variables in the regression model). To calculate the odds ratios of haplotypes with SSPG, participants with SSPG values >75% percentile were classed as cases (i.e. individuals with IR) and the others as controls (normal insulin sensitivity). We then randomly selected a case from a family with at least one case, and a control from a family that consisted only of controls. Odds ratios were calculated based on the unrelated case-control samples, with the major haplotype as the reference. For the conditional single-marker analysis, the most significant SNP in a gene was selected and added as an extra covariate in the tests for other SNPs in the same gene. Snp.plotter was used to calculate pairwise *r* ^*2*^ measures of linkage disequilibrium (LD) between SNPs and to generate LD plots [23].

We also included three lifestyle characteristics (physical activity, smoking, and alcohol intake) as covariates to address possible correlations with obesity and IR indices. The participants were classified as being as sedentary or non-sedentary based on standard criteria described in a previous study [17]. Briefly, the average numbers of hours spent on five levels of physical activities per day, including basal such as sleeping or lying down, sedentary such as sitting or standing, slight such as casual walking, moderate such as aerobic dancing, and heavy such as swimming, were obtained from questionnaires answered by the participants. A person was classified as sedentary if the person’s physical inactivity score, calculated as (hours of sedentary activity)/(24-hours of basal activity), was higher than 0.5. The participants were also asked about their smoking status and a participant was defined as either current- or non-smoking (non-smoking status included never- and ex-smoking subcategories). Moreover, the participants were asked about their status of alcohol consumption (i.e., beer, wine, sake and liquor consumption) and participants were classified as drinkers if they were self-reported drinkers, otherwise they were classified as being non-drinkers. We also tested interactions between lifestyle factors and the SNPs based on the interaction tests provided in PBAT. An SNP with a lifestyle factor interaction term was tested individually by PBAT while keeping the main effect of the SNP and covariates in the model. Moreover, the main effect of each SNP on the trait was tested in samples stratified by the status of each lifestyle factor. The tests were performed based on the GEE models with covariates on the stratified samples.

#### Multiple testing correction

We analyzed obesity and three IR-related measures (SSPG, FPI and HOMA-IR) for four SNPs, which resulted in 16 tests for single-marker analysis. Because the traits were correlated and the SNPs were in LD, using Bonferroni correction to adjust for the 16 tests would be conservative. Therefore, we used Monte Carlo simulations, which accounted for correlations among tests, to calculate the adjusted *p* values for multiple comparisons. We generated a population of haplotypes of the four SNPs based on the haplotype frequencies. Consequently, the simulated data had a LD structure similar to real data. The simulation software, SeqSIMLA [24], was then used to simulate 1,000 replicates of families with the same family structures to the real data based on the haplotypes. The real trait values were kept in the simulations so that the correlations among traits were preserved. For each replicate of families, 16 tests were performed based on PBAT, and the minimum *p* value from the 16 tests was selected. Taking the original *p* value for one of the 16 tests to be *p*_*i*_, the adjusted *p* value for *p*_*i*_ was calculated as the number of minimum *p* values from the 1,000 replicates less than *p*_*i*_ divided by 1,000 [25].

## Results

### Demographic and baseline characteristics, allele frequencies, and LD structure

**Fig 1:**
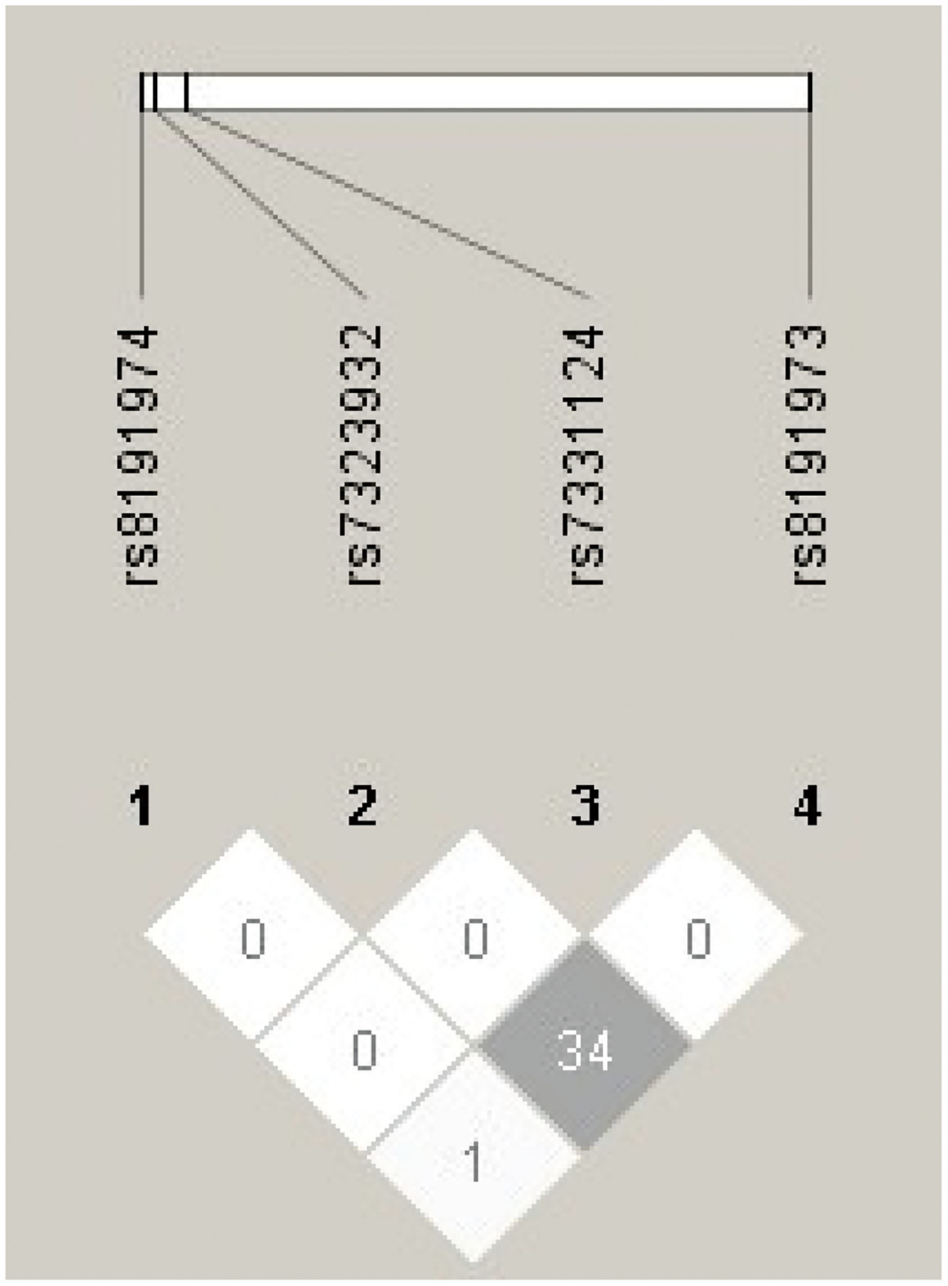
LD between *Gas6* SNPs in the study cohort. Two SNPs, rs7323932 and rs8191973, show moderate LD with *r* ^2^ = 0.34, while other SNP pairs are in linkage equilibrium.

Table 1 shows the basic clinical characteristics of the study participants at baseline and follow-up. A total of 984 participants (767 from the Chinese Han population) were included in the Phase I study, and 522 participants were enrolled in Phase II (i.e. the present study). The mean follow-up period was 5.7 years, and the mean age of the study population at Phase I was 49.5 years with a mean BMI of 25.5 kg/m^2^. Among all participants, 77.9% were Chinese, 65.2% had hypertension, and 9.9% were obese. Of the 984 participants enrolled in Phase I, SSPG was assessed in a subset of 221 individuals (summary statistics for this subset are shown in Table 1). When assessed at follow-up (i.e. Phase II), 16.7% of the 522 participants were found to have developed T2D (without evidence of T2D in Phase I). More detailed summary statistics for each SNP category are shown in S1 Table. The nucleic acid composition, gene region, minor allele frequencies, calling rate of genotype, and HWE test of the selected SNPs are summarized in S2 Table. The LD structure between SNPs is shown in Fig 1.

**Table 1:** Summary of characteristics in Phase I and Phase II studies.

	Phase I		Phase II
Characteristics	Total (984)	SSPG (221)	Total (522)
Age (years)	49.5 ± 8.8	47.4 ± 7.6	53.7 ± 8.8
Gender (male, %)	44.0	47.1	43.9
Ethnicity (Chinese, %)	77.9	98.6	100.0
BMI (kg/m^2^)	25.5 ± 3.72	25.0 ± 3.34	25.1 ± 3.25
SSPG (mg/dl)		168.0 ± 69.6	
FPG (mg/dl)	90.5 ± 10.8	88.9 ± 10.7	
FPI (uU/ml)	7.62 ± 6.03	7.38 ± 4.77	
HOMA-IR	0.99 ± 0.78	0.96 ± 0.62	
Hypertension (%)	65.2	64.9	
Obesity (%)	11.9	11.8	
Waist circumference	85.03 ± 10.99	85.02 ± 11.03	
Waist-hip ratio	0.87 ± 0.08	0.87 ± 0.08	
T2D (%)			16.7
Sedentary (%)	66.7	65.1	
Current smoking (%)	17.3	18.9	
Drinker (%)	28.7	29.3	

Data presented as mean ± standard deviation unless otherwise indicated. BMI: body mass index, DM: diabetes mellitus, FPG: fasting plasma glucose, FPI: fasting plasma insulin, HOMA-IR: homeostasis model assessment of insulin resistance, SSPG: steady-state plasma glucose, T2D: type 2 diabetes.

### SNP association with obesity and insulin resistance at baseline

The association analysis results between genetic variants of *Gas6* and obesity and IR indices are summarized in Table 2. The association *p* values, as well as the estimated odds ratios with 95% confidence intervals (CI) for obesity and the adjusted means (adjusted by covariates) with 95% CI for each continuous trait (SSPG, FPI and HOMA-IR) within each genotype category are shown in Table 2. Model 1 and Model 2 in Table 2 represent the models not including and including lifestyle factors in the covariates, respectively. For Model 1, none of the SNPs were associated with obesity. The association *p* values for waist circumference and waist-hip ratio are shown in S3 Table. The *p* values were not significant except for rs8191974 (which had an association *p* value of 0.032 for waist-hip ratio). However, three SNPs (rs8191973, rs8191974 and rs7323932) were associated with SSPG levels (*p* = 0.007, *p* = 0.03 and *p* = 0.011, respectively). A similar trend was seen when the Chinese Han population was analyzed separately (data not shown), however this association became non-significant for SNPs rs8191974 and rs7323932 when the analysis was performed conditional on rs8191973 (data not shown). The adjusted *p* values for multiple testing based on the Monte Carlo simulations are also shown in Table 2 for tests with *p* values < 0.05. The adjusted *p* value was 0.05 for the smallest *p* value (0.007), which was still significant after stringent correction. However, no other significant correlations were observed between *Gas6* polymorphisms and other indices of IR including HOMA-IR and FPI. For Model 2 that the three lifestyle factors were included as covariates, in general, the *p* values after adjusting for the lifestyle factors were similar to those without the adjustment in Model 1. The association of rs8191973 with SSPG remained significant (*p* = 0.007). Table 3 shows the interaction tests between the SNPs and the lifestyle factors. Interestingly, the SNP rs8191973 showed an interaction *p* value of 0.007 with physical activity, while the *p* value for the main effect of the SNP was 0.018 (Table 3). However, the interaction *p* value was not significant after adjusting for multiple interaction tests for the four SNPs and three lifestyle factors for four traits. Table 3 also shows the regression coefficients for the main effects of the SNPs in the stratified samples based on the lifestyle factors. The SNP rs8191973 had a negative effect on SSPG in sedentary samples, while the effect is positive in non-sedentary samples. The odds ratios of SNPs for obesity and the adjusted means for continuous traits in the stratified samples are shown in S4 Table. Table 4 summarizes specific haplotypes of four SNPs associated with SSPG and the extent of the associations with SSPG levels. Haplotype AACG (from rs8191974, rs7323932, rs7331124 and rs8191973) was positively associated with SSPG levels (*p* = 0.0098), and this association remained even after further adjusting for hypertension status (*p* = 0.012). The haplotype AACG had a higher odds ratio of 1.28 (95% confidence interval: 0.61, 2.64) when using the most prevalent GACG haplotype as a reference (data not shown).

**Table 2:** Association analysis results between the *Gas* 6 SNPs and obesity and insulin resistance indices.

SNP	Model	obesity^1^	SSPG^2^	FPI^2^ ^,^ ^3^	HOMA-IR^2^ ^,^ ^3^
rs8191974	Model 1^4^	0.859	0.030 (0.31^6^)	0.819	0.395
	Model 2^5^	0.556	0.044	0.694	0.370
GG	Model 1	reference	139.43 (123.64, 155.21)^7^	1.76 (1.67, 1.85)	-0.11 (-0.18, -0.04)
	Model 2	reference	140.71 (121.15, 160.27)	1.67 (1.57, 1.77)	-0.15 (-0.23, -0.07)
GA	Model 1	0.62 (0.37, 1.04)^8^	152.70 (136.49, 168.92)	1.77 (1.68, 1.87)	-0.13 (-0.21, -0.05)
	Model 2	0.52 (0.29, 0.93)	154.88 (134.24, 175.51)	1.69 (1.58, 1.80)	-0.17 (-0.26, -0.07)
AA	Model 1	1.16 (0.45. 2.98)	176.45 (146.04, 206.87)	1.75 (1.56, 1.94)	-0.13 (-0.28, 0.01)
	Model 2	1.00 (0.35, 2.88)	179.34 (145.61, 213.07)	1.65 (1.44, 1.85)	-0.17 (-0.33, -0.02)
rs7323932	Model 1	0.201	0.011 (0.11)	0.530	0.902
	Model 2	0.116	0.010	0.731	0.972
AA	Model 1	reference	149.55 (137.95, 161.16)	1.75 (1.65, 1.84)	-0.13 (-0.20, -0.05)
	Model 2	reference	150.35 (133.33, 167.37)	1.66 (1.56, 1.77)	-0.17 (-0.25, -0.08)
GA	Model 1	0.88 (0.55, 1.43)	139.01 (120.48, 157.54)	1.78 (1.69, 1.87)	-0.09 (-0.16, -0.01)
	Model 2	0.87 (0.54, 1.44)	140.15 (118.36, 161.93)	1.70 (1.59, 1.80)	-0.12 (-0.21, -0.04)
GG	Model 1	0.77 (0.31, 1.94)	161.14 (137.02, 185.27)	1.76 (1.62, 1.90)	-0.18 (-0.31, -0.05)
	Model 2	0.86 (0.33, 2.19)	162.27 (136.15, 188.39)	1.67 (1.53, 1.82)	-0.22 (-0.36, -0.08)
rs7331124	Model 1	0.585	0.911	0.164	0.509
	Model 2	0.554	0.871	0.193	0.551
CC	Model 1	reference	149.58 (139.87, 159.28)	1.76 (1.68, 1.85)	-0.12 (-0.19, -0.05)
	Model 2	reference	150.60 (135.75, 165.44)	1.68 (1.58, 1.77)	-0.16 (-0.24, -0.08)
TC	Model 1	1.04 (0.51, 2.11)	146.87 (126.93, 166.81)	1.78 (1.66, 1.90)	-0.10 (-0.20, 0.00)
	Model 2	1.12 (0.55, 2.29)	147.64 (122.88, 172.41)	1.70 (1.57, 1.84)	-0.13 (-0.24, -0.02)
TT	Model 1	NA^9^	NA	NA	NA
	Model 2	NA	NA	NA	NA
rs8191973	Model 1	0.508	0.007 (0.05)	0.610	0.763
	Model 2	0.662^4^	0.007	0.508	0.405
GG	Model 1	reference	153.55 (142.98, 164.11)	1.76 (1.67, 1.86)	-0.12 (-0.19, -0.05)
	Model 2	reference	154.37 (138.79, 169.96)	1.68 (1.58, 1.78)	-0.16 (-0.24, -0.08)
GC	Model 1	1.02 (0.62, 1.69)	138.78 (120.82, 156.74)	1.76 (1.67, 1.86)	-0.10 (-0.18, -0.03)
	Model 2	1.12 (0.67, 1.87)	138.95 (117.63, 160.26)	1.67 (1.56, 1.78)	-0.14 (-0.23, -0.05)
CC	Model 1	1.26 (0.45, 3.53)	132.37 (76.05, 188.68)	1.74 (1.56, 1.92)	-0.23 (-0.41, -0.05)
	Model 2	1.56 (0.55, 4.46)	135.61 (77.89, 193.32)	1.71 (1.50, 1.92)	-0.21 (-0.42, 0.00)

SSPG: steady state plasma glucose, FPI: fasting plasma insulin, HOMA-IR: homeostasis model assessment of insulin resistance.

^1^ Model adjusted for age, gender, site, and ethnic population.

^2^ Model adjusted for age, gender, site, BMI, and ethnic population.

^3^ Analysis with log transformation

^4^ Model does not include lifestyle factors as covariates

^5^ Model includes lifestyle factors as covariates

^6^ Adjusted p-value for multiple testing

^7^ Adjusted means and 95% CI

^8^ Odds ratios and 95% CI

^9^ No data in the category

**Table 3:** Interaction tests between SNPs and lifestyle factors.

SNP		Obesity^1^	SSPG^2^	FPI^2^ ^,^ ^3^	HOMA-IR^2^ ^,^ ^3^
rs8191974					
Physical activity	Sedentary	0.33±0.28^4^	-6.54±10.58	0.006±0.05	0.01±0.04
	Non-sedentary	0.60±0.42	-6.19±13.29	0.08±0.07	0.10±0.05
	P value^5^	0.556	0.102	0.767	0.327
Smoking	Yes	0.72±0.50	-33.72±18.55	-0.03±0.11	0.06±0.08
	No	0.38±0.28	1.51±9.99	0.04±0.04	0.04±0.03
	P value	0.783	0.195	0.845	0.997
Alcohol consumption	Yes	0.55±0.44	-5.27±16.37	0.03±0.07	0.05±0.06
	No	0.29±0.30	-4.26±10.50	0.04±0.04	0.04±0.03
	P value	0.599	0.150	0.559	0.346
rs7323932					
Physical activity	Sedentary	0.03±0.22	-0.48±9.09	0.002±0.04	-0.01±0.04
	Non-sedentary	-0.34±0.36	12.27±12.82	0.06±0.06	0.08±0.06
	P value	0.077	0.017	0.528	0.708
Smoking	Yes	-0.30±0.52	-7.31±12.94	0.04±0.08	-0.03±0.07
	No	-0.12±0.22	-0.14±7.89	0.01±0.04	0.01±0.03
	P value	0.027	0.011	0.862	0.980
Alcohol consumption	Yes	-0.09±0.33	-10.57±12.74	0.01±0.07	-0.04±0.06
	No	-0.08±0.24	7.34±8.47	0.02±0.04	0.03±0.03
	P value	0.056	0.022	0.595	0.780
rs7331124					
Physical activity	Sedentary	0.36±0.43	-7.59±15.81	-0.004±0.07	-0.002±0.07
	Non-sedentary	-0.44±0.85	-1.62±25.13	0.05±0.11	0.06±0.09
	P value	0.700	0.962	0.073	0.391
Smoking	Yes	0.88±0.95	-43.16±12.48	0.12±0.14	0.12±0.14
	No	-0.12±0.43	6.22±12.34	-0.02±0.06	-0.02±0.05
	P value	0.885	0.253	0.110	0.787
Alcohol consumption	Yes	0.61±0.76	-5.26±21.88	0.44±0.15	0.36±0.13
	No	0.03±0.42	-6.24±15.75	-0.07±0.06	-0.05±0.06
	P value	0.474	0.881	0.344	0.759
rs8191973					
Physical activity	Sedentary	0.24±0.26	-24.40±11.68	0.03±0.06	0.02±0.05
	Non-sedentary	0.11±0.35	22.17±24.36	0.02±0.08	0.04±0.07
	P value	0.764	0.007	0.366	0.327
Smoking	Yes	0.18±0.92	17.06±31.53	0.06±0.10	0.10±0.09
	No	0.17±0.25	-5.86±11.75	0.01±0.05	0.008±0.04
	P value	0.349	0.112	0.708	0.680
Alcohol consumption	Yes	0.25±0.39	-43.00±15.27	-0.04±0.09	0.003±0.08
	No	0.14±0.26	-6.30±14.47	0.05±0.05	0.02±0.04
	P value	0.896	0.017	0.419	0.255

^1^ Model adjusted for age, gender, site, ethnic population, physical activity, smoking, and alcohol consumption.

^2^ Model adjusted for age, gender, region, BMI, ethnic population, physical activity, smoking, and alcohol consumption.

^3^ Analysis with log transformation.

^4^ β coefficients and standard errors for the main effects of SNPs.

^5^ Interaction p-values.

BMI: body mass index, FPI: fasting plasma insulin, HOMA-IR: homeostasis model assessment of insulin resistance, SNP: single nucleotide polymorphism, SSPG: steady-state plasma glucose.

**Table 4:** Haplotype analysis of *Gas6* SNPs and SSPG.

rs8191974	rs7323932	rs7331124	rs8191973	Frequency	*p* value
G	A	C	G	0.4258	0.8567
A	A	C	G	0.1541	0.0098
G	G	C	G	0.1109	0.8499
G	G	C	C	0.1029	0.0429
G	A	T	G	0.0670	0.5958
A	G	C	G	0.0502	0.4187
G	A	C	C	0.0185	0.1763
G	G	T	C	0.0156	0.3282
A	G	C	C	0.0127	0.3126
A	A	C	C	0.0120	0.4977
G	G	T	G	0.0117	0.2057
A	G	T	G	0.0076	0.3307
A	A	T	G	0.0075	0.7473
G	A	T	C	0.0035	0.4060
Overall					0.2085

SNP: single nucleotide polymorphism, SSPG: steady-state plasma glucose.

All of the analyses were performed based on the assumption of an additive model. We also performed association analysis for obesity and IR indices using dominant and recessive models (S5 Table). The *p* values for rs8191973 became less significant with SSPG (0.022 in the dominant and 0.067 in the recessive models). This suggests that the SNP is more likely to have an additive effect on SSPG. In contrast, the *p* value for rs7323932 became more significant (0.005) in the dominant model than in the additive model (0.011).

We then calculated the power for testing a single-marker association between the SNPs and SSPG, and the power for testing the association of haplotypes with SSPG using a bootstrap procedure [26]. Families were sampled with replacement of each bootstrap replicate, and an association test was performed on the bootstrap replicate. Power was calculated based on the proportion of tests with *p* values < 0.05 in 1,000 bootstrap replicates. The power values for testing rs8191974, rs7323932, rs7331124 and rs8191973 with SSPG were 0.569, 0.682, 0.043 and 0.796, respectively. Therefore, our study design had reasonable power to detect the association between rs8191973 and SSPG. The power for testing the association between the AACG haplotype at the SNPs and SSPG was 0.138. Thus, the study results should be interpreted cautiously under the relatively low power, and further studies are needed to validate our findings.

### SNP association with the incidence of type 2 diabetes

A total of 522 participants were evaluated for an average follow-up period of 5.7 years, to further explore the role of the *Gas6* gene in the development of T2D. None of the polymorphisms were associated with an increased risk for T2D development after adjusting for age, gender, BMI, region, lifestyle factors, and ethnic factors, with *p* values of 0.773, 0.296, 0.897 and 0.678 for rs8191973, rs8191974, rs7323932 and rs7331124, respectively.

## Discussion

In this study, we demonstrated that a *Gas6* genetic variant was associated with IR in an Asian cohort of hypertensive patients. Although the effects of these genetic variants on subsequent progression to T2D remain unclear, our previous findings suggest that plasma Gas6 levels may protect against T2D, elevated FPG, inflammation, and endothelial dysfunction [11]. These findings suggest a possible link between *Gas6* genetic variants and IR.

In the present study, we found that none of the *Gas6* genetic SNPs had a positive association with obesity. Although our results were not significant, data based on an animal model suggest that the Gas/TAM system plays a vital role in adipose tissue accumulation and body weight regulation [27–29]. Gas6 expression in subcutaneous fat was increased when fed with high-fat diet mice as comparing to those fed with a standard diet [27]. Similarly, Maquoi E et al observed that Gas6-deficient mice had significantly less fat mass than their wild-type counterparts when exposed to a high-fat diet [28]. In a murine model of nutritionally induced obesity, Lijnen HR et al found Gas6 signaling will be affected via receptor antagonsim and therefore impaired adipocte differentiation and reduced adipose tissue development [29]. We also recently confirmed the close association between Gas6 and obesity and Gas6 and inflammation in both adults and adolescents [11], [12]. Our results are not consistent with another recent study, in which adolescent boys, but not girls, with the GG genotype of *Gas6* rs8191973 and rs8191974, exhibited higher adiposity [30]. This discrepancy may be due to several factors including age, different adiposity criteria, and sex hormone profiles. Although serum Gas6 protein levels were not measured in the present study, Hsiao and colleagues observed a lack of positive association between *Gas6* genotypes and circulating Gas6 proteins levels [30]. Taken together, it implies that Gas6 signaling may play an important role in the development of obesity, but the exact mechanism of Gas6 and its genetic polymorphisms on adiposity is unknown, and further investigations are needed.

The major pathophysiological mechanism in T2D is IR. Our results suggest that the *Gas6* genetic variant rs8191973 plays an important role in the development of IR. Using the gold standard IST method to measure IR, the present study is the first to explore the association between *Gas6* genetic variants and IR indices and their role in the development of T2D. Our results showed that the SNP rs8191973 but not rs8191974 had a significant association with SSPG in the additive model. This finding is in contrast to previous studies, in which IR did not differ between genotypes of rs8191973 and rs8191974 [15], [30]. The different methodologies used to measure IR most likely explains this discrepancy (as the IST method used in the present study is the gold standard and therefore more reliable). Variations between study populations, including age, may also explain the different results. Similar to the findings from our previous study, the SSPG levels of different genotypes among the four SNPs were not significantly different in either recessive or dominant modes [15]. The Gas6/TAM system may involve the important pathological mechanism of IR through targeting inflammation. Pro-inflammatory cytokines, such as tumor necrosis factor-α (TNF-α) and interleukin 6 (IL-6), contribute to IR by triggering key steps in the insulin signal pathway [31]. In cell study, Gas6 and Mer receptor can modulate macrophage secretion of cytokines, including TNF-α and IL-6 to exhibit its anti-inflammatory effect [32]. Furthermore, in another animal study, Gas6-deficient mice displayed reduction of hepatic inflammation, revealed by decreased expression of pro-inflammatory cytokines, including TNF-α [33]. Our previous study demonstrated that plasma Gas6 levels are associated with glucose intolerance and markers of inflammation both in adults and adolescents [11], [12]. The present study, taken together with clinical and pre-clinical evidence, may indicate that genetic variants of *Gas6* play a role in IR; however, a large-scale study is needed to validate this.

Although the results of this study support a possible genetic contribution to IR, the contribution of the *Gas6* gene in the subsequent development of T2D appears minimal. The present findings also in contrast to our previous study which demonstrated a lower frequency of the AA genotype of rs8191974 in T2D [15]. A possible explanation for this may be that variations in the *Gas6* gene may affect the risk of T2D via mechanisms that go beyond IR. The genetic contribution to the heritability of T2D has been estimated to be approximately 10% [34], and therefore caution should be exercised when considering the role of *Gas6* gene variations in the development of T2D. In particular, further studies may be necessary to explore the association between Gas6 protein regulation and *Gas6* genetic variants.

Insulin resistance has a complex and heterogeneous genetic background that also involves the contribution of environmental factors. In this study, we explored the interaction between lifestyle and genetic factors in influencing SSPG levels, and we found that the association between rs8191973 and SSPG remained significant after adjusting for lifestyle factors. Interestingly, the SNP rs8191973 also appeared to interact with physical activity for SSPG. with an opposed effect of different levels physical activity. This suggests that the levels of physical activity may play an important contribution of *Gas6* genetic variants on IR. However, a large, prospective study is needed to confirm this relationship.

A major strength of the present study is that we used the gold standard method of measuring IR, IST. Moreover, this is the first prospective study to evaluate the genetic role of *Gas6* on the risk of developing T2D. However, the present study also has several limitations. First, the number of study participants was relatively small (which may not provide enough statistical power to detect significant associations). Second, circulating Gas6 protein levels were not measured (which would have helped in the interpretation of the results of genetic analysis). Third, gene-gene and gene-environmental interactions were not analyzed.

## Conclusions

In brief, we conclude based on the present findings that *Gas6* gene variants are associated with IR in the Chinese population. However, the genetic contribution of *Gas6* to the risk for T2D should be further explored in a large, prospective study.

## Software Used for Statistical Analysis

PBAT (http://www.hsph.harvard.edu/clange/default.htm): single-marker association test, haplotype association test, interaction association test

Snp.plotter: An R package for generating LD plots (Fig 1)

Geepack package in R: An R package for GEE regression

Lsmeans package in R: An R package for calculating the adjusted means in Table 2

## Supporting Information

S1 Table Summary of characteristics in Phase I and Phase II studies against genotypes. (DOCX)

S2 Table The nucleic acid composition, gene region, minor allele frequencies, calling rate of genotype and Hardy-Weinberg equilibrium test of selected SNPs. (DOCX)

S3 Table Association analysis p-values between Gas6 SNPs and waist-circumference and waist-hip-ratio. (DOCX)

S4 Table Odds ratios and adjusted means for the interaction model. (DOCX)

S5 Table Association analysis p-values between *Gas* 6 SNPs, obesity, and insulin resistance indices using dominant and recessive models. (DOCX)
