# Supplementary material for: Effect of Common Genetic Variants of Growth Arrest-Specific 6 Gene on Insulin Resistance, Obesity and Type 2 Diabetes in an Asian Population
Source: PLoS One. 2015 Aug 18;10(8):e0135681. doi: 10.1371/journal.pone.0135681 (PMC4540485; doi:10.1371/journal.pone.0135681)
Supplement: S1 Table — (DOCX) [file pone.0135681.s001.docx]

**S1 Table.**

|  | Phase I | | Phase II |
| --- | --- | --- | --- |
| Characteristics | Total (984) | SSPG (221) | Total (522) |
| Age (year) | 49.5±8.8 | 47.4±7.6 | 53.7±8.8 |
| rs8191974 | (970) | (220) | (519) |
| GG | 49.7±8.7 | 46.9±6.9 | 53.9±8.6 |
| GA | 49.1±9.1 | 48.0±8.6 | 53.3±9.1 |
| AA | 48.2±6.9 | 48.6±8.0 | 50.3±6.6 |
| rs7323932 | (978) | (220) | (520) |
| GG | 50.0±8.2 | 46.5±5.3 | 56.1±8.5 |
| GA | 48.5±8.8 | 46.5±7.7 | 52.2±8.7 |
| AA | 50.1±8.7 | 48.0±7.8 | 54.3±8.6 |
| rs7331124 | (984) | (221) | (522) |
| TT | 44.2±8.1 | 39.0±5.6 | 51.6±8.8 |
| TC | 47.2±7.9 | 46.2±7.2 | 51.5±7.2 |
| CC | 49.8±8.8 | 47.7±7.6 | 54.1±9.0 |
| rs8191973 | (976) | (219) | (516) |
| GG | 49.5±8.5 | 47.8±7.7 | 53.4±8.7 |
| GC | 49.5±9.3 | 46.0±7.0 | 54.0±8.9 |
| CC | 48.8±7.5 | 49.0±1.4 | 53.9±7.0 |
| Gender (male, %) | 44.0 | 47.1 | 43.9 |
| rs8191974 | 43.9 | 47.2 | 43.7 |
| GG | 42.8 | 43.5 | 42.6 |
| GA | 47.4 | 54.2 | 47.2 |
| AA | 36.3 | 38.4 | 31.2 |
| rs7323932 | 44.1 | 47.2 | 43.8 |
| GG | 46.1 | 57.1 | 45.9 |
| GA | 38.7 | 42.8 | 38.4 |
| AA | 48.0 | 48.8 | 47.5 |
| rs7331124 | 44.0 | 47.0 | 43.8 |
| TT | 80.0 | 100.0 | 100.0 |
| TC | 45.3 | 68.4 | 42.4 |
| CC | 43.5 | 41.9 | 43.6 |
| rs8191973 | 43.9 | 47.0 | 43.6 |
| GG | 45.8 | 45.7 | 45.7 |
| GC | 39.8 | 52.3 | 38.2 |
| CC | 36.0 | 50.0 | 38.4 |
| Ethnic (Chinese, %) | 77.9 | 98.6 | 100.0 |
| rs8191974 | 77.8 | 98.6 |  |
| GG | 75.0 | 99.1 |  |
| GA | 82.6 | 98.7 |  |
| AA | 86.3 | 92.3 |  |
| rs7323932 | 78.0 | 98.6 |  |
| GG | 78.4 | 92.8 |  |
| GA | 79.2 | 100.0 |  |
| AA | 77.0 | 98.4 |  |
| rs7331124 | 77.9 | 98.6 |  |
| TT | 100.0 | 100.0 |  |
| TC | 94.5 | 100.0 |  |
| CC | 75.3 | 98.3 |  |
| rs8191973 | 77.8 | 98.6 |  |
| GG | 78.5 | 98.8 |  |
| GC | 76.3 | 97.6 |  |
| CC | 76.0 | 100.0 |  |
| BMI (kg/m^2^) | 25.5±3.72 | 25.0±3.34 | 25.1±3.25 |
| rs8191974 | (817) | (194) | (475) |
| GG | 25.7±3.8 | 25.3±3.5 | 25.8±3.5 |
| GA | 25.1±3.3 | 24.9±3.1 | 25.5±3.2 |
| AA | 25.5±3.5 | 24.8±3.0 | 25.2±4.1 |
| rs7323932 | (815) | (193) | (476) |
| GG | 25.1±4.5 | 24.2±2.3 | 24.6±3.4 |
| GA | 25.4±3.5 | 25.6±3.5 | 25.5±3.3 |
| AA | 25.6±3.7 | 24.9±3.3 | 26.0±3.5 |
| rs7331124 | (820) | (194) | (478) |
| TT | 23.5±2.2 | 21.2±2.3 | 26.3±3.7 |
| TC | 25.2±3.5 | 24.7±2.4 | 25.4±3.5 |
| CC | 25.5±3.7 | 25.2±3.3 | 25.8±3.4 |
| rs8191973 | (814) | (192) | (472) |
| GG | 25.5±3.6 | 25.1±3.2 | 25.8±3.5 |
| GC | 25.4±3.6 | 25.2±3.6 | 25.5±3.3 |
| CC | 25.7±5.8 | 23.8±3.3 | 23.7±3.9 |
| SSPG (mg/dl) |  | 168.0±69.6 |  |
| rs8191974 |  | (220) |  |
| GG |  | 166.7±74.0 |  |
| GA |  | 169.0±61.8 |  |
| AA |  | 168.1±79.5 |  |
| rs7323932 |  | (220) |  |
| GG |  | 161.7±60.6 |  |
| GA |  | 169.7±76.3 |  |
| AA |  | 167.5±67.0 |  |
| rs7331124 |  | (221) |  |
| TT |  | 177.2±93.9 |  |
| TC |  | 169.2±58.7 |  |
| CC |  | 167.6±71.8 |  |
| rs8191973 |  | (219) |  |
| GG |  | 170.4±65.9 |  |
| GC |  | 160.9±85.3 |  |
| CC |  | 121.2±22.6 |  |
| FPG (mg/dl) | 90.5±10.8 | 88.9±10.7 |  |
| rs8191974 | (953) | (220) |  |
| GG | 90.8±10.7 | 88.7±1.4 |  |
| GA | 89.6±10.9 | 88.5±9.6 |  |
| AA | 92.1±10.3 | 92.7±10.0 |  |
| rs7323932 | (951) | (220) |  |
| GG | 90.1±10.0 | 87.6±7.5 |  |
| GA | 90.6±11.0 | 90.7±11.4 |  |
| AA | 90.3±10.7 | 87.9±10.6 |  |
| rs7331124 | (957) | (221) |  |
| TT | 84.7±5.8 | 88.2±7.4 |  |
| TC | 88.9±11.9 | 86.6±10.6 |  |
| CC | 90.7±10.6 | 89.4±10.7 |  |
| rs8191973 | (949) | (219) |  |
| GG | 90.4±11.2 | 89.1±11.2 |  |
| GC | 90.8±9.6 | 88.4±8.4 |  |
| CC | 87.0±8.8 | 79.5±10.6 |  |
| FPI (uU/ml) | 7.62±6.03 | 7.38±4.77 |  |
| rs8191974 | (950) | (219) |  |
| GG | 7.89±6.7 | 7.58±5.5 |  |
| GA | 7.07±4.4 | 7.15±3.4 |  |
| AA | 7.42±4.1 | 6.36±3.9 |  |
| rs7323932 | (948) | (219) |  |
| GG | 7.24±7.4 | 5.37±1.7 |  |
| GA | 7.69±5.4 | 8.26±5.4 |  |
| AA | 7.64±6.2 | 7.06±4.5 |  |
| rs7331124 | (954) | (220) |  |
| TT | 7.24±7.4 | 5.25±4.4 |  |
| TC | 7.69±5.4 | 6.77±2.8 |  |
| CC | 7.64±6.2 | 7.52±5.0 |  |
| rs8191973 | (946) | (218) |  |
| GG | 7.64±5.9 | 7.31±4.3 |  |
| GC | 7.48±5.5 | 7.87±6.3 |  |
| CC | 8.37±11.2 | 5.15±2.3 |  |
| HOMA-IR | 0.99±0.78 | 0.96±0.62 |  |
| rs8191974 | (846) | (198) |  |
| GG | 1.09±0.7 | 1.09±0.7 |  |
| GA | 0.99±0.5 | 0.95±0.4 |  |
| AA | 1.01±0.4 | 0.96±0.4 |  |
| rs7323932 | (844) | (198) |  |
| GG | 1.00±0.9 | 0.68±0.2 |  |
| GA | 1.09±0.6 | 1.17±0.6 |  |
| AA | 1.05±0.6 | 0.98±0.5 |  |
| rs7331124 | (849) | (199) |  |
| TT | 0.66±0.3 | 1.10±0 |  |
| TC | 1.05±0.6 | 0.95±0.3 |  |
| CC | 1.05±0.7 | 1.04±0.6 |  |
| rs8191973 | (843) | (198) |  |
| GG | 1.05±0.6 | 1.01±0.5 |  |
| GC | 1.06±0.7 | 1.15±0.8 |  |
| CC | 1.13±1.4 | 0.65±0.3 |  |
| Hypertension (%) | 65.2 | 64.9 |  |
| rs8191974 | 65.2 | 64.8 |  |
| GG | 64.8 | 63.8 |  |
| GA | 66.0 | 67.6 |  |
| AA | 64.8 | 58.3 |  |
| rs7323932 | 65.2 | 65.1 |  |
| GG | 69.8 | 78.5 |  |
| GA | 59.4 | 60.5 |  |
| AA | 69.2 | 66.3 |  |
| rs7331124 | 65.2 | 64.8 |  |
| TT | 75.0 | 100.0 |  |
| TC | 58.8 | 63.6 |  |
| CC | 66.0 | 64.8 |  |
| rs8191973 | 65.2 | 65.0 |  |
| GG | 66.4 | 66.4 |  |
| GC | 61.6 | 57.5 |  |
| CC | 68.1 | 100.0 |  |
| Obesity (%) | 11.9 | 11.8 |  |
| rs8191974 | 11.9 | 11.8 |  |
| GG | 13.7 | 14.9 |  |
| GA | 7.8 | 7.3 |  |
| AA | 13.5 | 8.3 |  |
| rs7323932 | 12.0 | 11.9 |  |
| GG | 9.4 | 0 |  |
| GA | 11.4 | 17.6 |  |
| AA | 12.8 | 9.9 |  |
| rs7331124 | 11.9 | 11.8 |  |
| TT | 0 | 0 |  |
| TC | 10.7 | 10.0 |  |
| CC | 12.1 | 12.2 |  |
| rs8191973 | 11.9 | 11.9 |  |
| GG | 11.8 | 10.6 |  |
| GC | 11.9 | 17.5 |  |
| CC | 14.2 | 0 |  |
| Waist-circumference | 85.03±10.99 | 85.02±11.03 |  |
| rs8191974 | (815) | (194) |  |
| GG | 85.32±11.1 | 83.15±10.4 |  |
| GA | 84.30±10.2 | 83.41±9.7 |  |
| AA | 86.28±12.6 | 86.683±10.9 |  |
| rs7323932 | (813) | (193) |  |
| GG | 83.27±11.7 | 81.96±10.4 |  |
| GA | 84.61±10.7 | 84.17±11.3 |  |
| AA | 85.68±11.0 | 83.36±9.4 |  |
| rs7331124 | (818) | (194) |  |
| TT | 75.00±4.9 | 75.00±0 |  |
| TC | 82.64±10.0 | 83.29±9.2 |  |
| CC | 85.43±11.0 | 83.54±10.4 |  |
| rs8191973 | (812) | (192) |  |
| GG | 85.46±11.0 | 83.75±10.1 |  |
| GC | 84.21±10.3 | 83.12±10.5 |  |
| CC | 83.61±14.4 | 80.50±14.8 |  |
| Waist-hip-ratio | 0.87±0.08 | 0.87±0.08 |  |
| rs8191974 | (815) | (194) |  |
| GG | 0.88±0.08 | 0.85±0.07 |  |
| GA | 0.87±0.08 | 0.86±0.07 |  |
| AA | 0.88±0.1 | 0.91±0.1 |  |
| rs7323932 | (813) | (193) |  |
| GG | 0.86±0.08 | 0.84±0.08 |  |
| GA | 0.87±0.08 | 0.85±0.07 |  |
| AA | 0.88±0.08 | 0.86±0.07 |  |
| rs7331124 | (818) | (194) |  |
| TT | 0.78±0.02 | 0.78±0 |  |
| TC | 0.85±0.07 | 0.87±0.07 |  |
| CC | 0.88±0.08 | 0.86±0.07 |  |
| rs8191973 | (812) | (192) |  |
| GG | 0.88±0.08 | 0.86±0.07 |  |
| GC | 0.86±0.08 | 0.84±0.07 |  |
| CC | 0.86±0.08 | 0.85±0.1 |  |
| DM (%) |  |  | 16.7 |
| rs8191974 |  |  | 16.1 |
| GG |  |  | 16.6 |
| GA |  |  | 14.5 |
| AA |  |  | 20.0 |
| rs7323932 |  |  | 16.3 |
| GG |  |  | 16.6 |
| GA |  |  | 16.0 |
| AA |  |  | 16.4 |
| rs7331124 |  |  | 16.2 |
| TT |  |  | 25.0 |
| TC |  |  | 14.6 |
| CC |  |  | 16.5 |
| rs8191973 |  |  | 16.2 |
| GG |  |  | 15.7 |
| GC |  |  | 18.1 |
| CC |  |  | 9.0 |
| Sedentary (%) |  |  |  |
| rs8191974 | 66.5 | 65.1 |  |
| GG | 66.8 | 62.9 |  |
| GA | 64.1 | 67.6 |  |
| AA | 77.7 | 72.7 |  |
| rs7323932 | 66.5 | 64.9 |  |
| GG | 67.9 | 85.7 |  |
| GA | 65.9 | 67.7 |  |
| AA | 66.8 | 60.7 |  |
| rs7331124 | 66.7 | 65.1 |  |
| TT | 100.0 | 100.0 |  |
| TC | 68.6 | 66.6 |  |
| CC | 66.1 | 64.6 |  |
| rs8191973 | 66.5 | 64.7 |  |
| GG | 65.7 | 62.9 |  |
| GC | 69.9 | 70.0 |  |
| CC | 52.3 | 100.0 |  |
| Smoking (%) |  |  |  |
| rs8191974 | 17.1 | 18.9 |  |
| GG | 17.0 | 18.9 |  |
| GA | 18.4 | 22.0 |  |
| AA | 11.1 | 0.0 |  |
| rs7323932 | 17.1 | 19.0 |  |
| GG | 18.8 | 28.5 |  |
| GA | 16.2 | 17.6 |  |
| AA | 17.8 | 18.7 |  |
| rs7331124 | 17.3 | 18.9 |  |
| TT | 75.0 | 100.0 |  |
| TC | 20.5 | 36.6 |  |
| CC | 16.5 | 15.2 |  |
| rs8191973 | 17.0 | 18.6 |  |
| GG | 17.4 | 18.5 |  |
| GC | 15.4 | 20.0 |  |
| CC | 23.8 | 0.0 |  |
| Drinker (%) |  |  |  |
| rs8191974 | 28.8 | 29.3 |  |
| GG | 28.1 | 26.3 |  |
| GA | 29.7 | 34.8 |  |
| AA | 32.3 | 27.7 |  |
| rs7323932 | 28.7 | 29.4 |  |
| GG | 34.0 | 42.8 |  |
| GA | 26.9 | 26.4 |  |
| AA | 29.4 | 29.6 |  |
| rs7331124 | 28.6 | 29.3 |  |
| TT | 25.0 | 100.0 |  |
| TC | 24.0 | 42.8 |  |
| CC | 29.3 | 26.5 |  |
| rs8191973 | 28.6 | 29.1 |  |
| GG | 27.7 | 30.6 |  |
| GC | 22.7 | 22.5 |  |
| CC | 31.0 | 50.0 |  |

Data are presented as Mean±SD unless otherwise indicated.

BMI: body mass index, SSPG: steady state plasma glucose, FPG: fasting plasma glucose, FPI: fasting plasma insulin, HOMA-IR: homeostasis model assessment of insulin resistance, DM: diabetes mellitus.
