# Supplementary material for: Effect of Common Genetic Variants of Growth Arrest-Specific 6 Gene on Insulin Resistance, Obesity and Type 2 Diabetes in an Asian Population
Source: PLoS One. 2015 Aug 18;10(8):e0135681. doi: 10.1371/journal.pone.0135681 (PMC4540485; doi:10.1371/journal.pone.0135681)
Supplement: S2 Table — (DOCX) [file pone.0135681.s002.docx]

**S2 Table.**

| Name | Minor/  Major allele | Gene Region | MAF | % call rate | HWE-P |
| --- | --- | --- | --- | --- | --- |
| rs8191973 | C/G | Intron 3 | 0.163 | 0.991 | 0.577 |
| rs8191974 | A/G | Intron 8, close to splice site sequence | 0.197 | 0.996 | 0.226 |
| rs7323932 | G/A | Intron 7 | 0.267 | 0.994 | 0.719 |
| rs7331124 | T/C | Intron 7 | 0.076 | 1 | 0.635 |

MAF: minor allele frequencies

HWE-P: Hardy-Weinberg equilibrium test p-values
