# Supplementary material for: Effect of Common Genetic Variants of Growth Arrest-Specific 6 Gene on Insulin Resistance, Obesity and Type 2 Diabetes in an Asian Population
Source: PLoS One. 2015 Aug 18;10(8):e0135681. doi: 10.1371/journal.pone.0135681 (PMC4540485; doi:10.1371/journal.pone.0135681)
Supplement: S3 Table — (DOCX) [file pone.0135681.s003.docx]

**S3 Table.**

| SNP | waist-circumference | waist-hip-ratio |
| --- | --- | --- |
| rs8191974 | 0.185 | 0.032 |
| rs7323932 | 0.103 | 0.281 |
| rs7331124 | 0.259 | 0.079 |
| rs8191973 | 0.560 | 0.405 |
