# Supplementary material for: Effect of Common Genetic Variants of Growth Arrest-Specific 6 Gene on Insulin Resistance, Obesity and Type 2 Diabetes in an Asian Population
Source: PLoS One. 2015 Aug 18;10(8):e0135681. doi: 10.1371/journal.pone.0135681 (PMC4540485; doi:10.1371/journal.pone.0135681)
Supplement: S4 Table — (DOCX) [file pone.0135681.s004.docx]

**S4 Table.**

| SNP |  | Obesity^1^ | SSPG^2^ | FPI^2,3^ | HOMA-IR^2,3^ |
| --- | --- | --- | --- | --- | --- |
| rs8191974 |  |  |  |  |  |
| Physical activity | Sedentary |  |  |  |  |
|  | GG | reference | 131.95 (103.58 , 160.32) | 1.72 (1.59 , 1.86) | -0.11 (-0.21 , -0.11) |
|  | GA | 0.43 (0.20 , 0.89) | 137.65 (111.14 , 164.16) | 1.74 (1.60 , 1.89) | -0.13 (-0.24,-0.02) |
|  | AA | 1.26 (0.44 , 3.64) | 164.07 (133.88 , 194.25) | 1.72 (1.46 , 1.98) | -0.07 (-0.27 , 0.13) |
|  | Non-sedentary |  |  |  |  |
|  | GG | reference | 139.97 (111.68 , 168.26) | 1.64 (1.44 , 1.83) | -0.19 (-0.35 , -0.02) |
|  | GA | 0.65 (0.25 , 1.69) | 168.42 (142.70 , 194.15) | 1.65 (1.43 , 1.86) | -0.19 (-0.39 , 0.01) |
|  | AA | NA^4^ | 141.54 (59.61 , 223.47) | 1.63 (1.39 , 1.86) | -0.35 (-0.57 , -0.14) |
| Smoking | Current Smoking |  |  |  |  |
|  | GG | reference | 146.64 (123.72 , 159.57) | 1.20 (1.01 , 1.38) | -0.58 (-0.77 , -0.39) |
|  | GA | 0.56 (0.17 , 1.84) | 180.74 (154.68 , 206.79) | 1.27 (1.02 , 1.53) | -0.57 (-0.83 , -0.31) |
|  | AA | NA | NA | 1.43 (1.25 , 1.62) | -0.68 (-0.87 , -0.49) |
|  | Non-smoking |  |  |  |  |
|  | GG | reference | 142.49 (125.11 , 159.87) | 1.77 (1.67 , 1.87) | -0.11 (-0.19 , -0.03) |
|  | GA | 0.44 (0.22 , 0.87) | 148.29 (128.40 , 168.18) | 1.77 (1.67 , 1.88) | -0.14 (-0.23 , -0.05) |
|  | AA | 1.21 (0.40 , 3.61) | 174.87 (140.44 , 209.29) | 1.75 (1.53 , 1.97) | -0.12 (-0.29 , 0.05) |
| Alcohol consumption | Drinker |  |  |  |  |
|  | GG | reference | 132.95 (109.21 , 156.69) | 1.71 (1.54 , 1.89) | -0.05 (-0.22 , 0.12) |
|  | GA | 0.26 (0.09 , 0.82) | 153.50 (128.05 , 178.95) | 1.81 (1.62 , 2.00) | -0.01 (-0.21 , 0.19) |
|  | AA | 1.46 (0.36 , 3.61) | 179.50 (126.23 , 232.78) | 1.73 (1.46 , 1.99) | -0.14 9-0.39 , 0.12) |
|  | Non-drinker |  |  |  |  |
|  | GG | reference | 152.28 (124.01 , 180.56) | 1.62 (1.46 , 1.78) | -0.18 (-0.30 , -0.07) |
|  | GA | 0.64 (0.31 , 1.33) | 158.95 (130.72 , 187.18) | 1.61 (1.44 , 1.78) | -0.22 (-0.36 , -0.09) |
|  | AA | 0.93 (0.20 , 4.28) | 181.13 (132.82 , 229.44) | 1.53 (1.21 , 1.84) | -0.20 (-0.41 , 0.02) |
|  |  |  |  |  |  |
|  |  |  |  |  |  |
|  |  |  |  |  |  |
| rs7323932 |  |  |  |  |  |
| Physical activity | Sedentary |  |  |  |  |
|  | AA | reference | 147.90 (121.77 , 174.03) | 1.73 91.57 , 1.88) | -0.11 (-0.22 , -0.01) |
|  | GA | 1.05 (0.60 , 1.83) | 127.12 (100.93 , 153.31) | 1.73 (1.59 , 1.86) | -0.10 (-0.20 , 0.01) |
|  | GG | 1.03 (0.33 , 3.23) | 154.68 (124.29 , 185.06) | 1.76 (1.59 , 1.94) | -0.19 9-0.36 , -0.02) |
|  | Non-sedentary |  |  |  |  |
|  | AA | reference | 152.14 (127.07 , 177.20) | 1.60 (1.40 , 1.79) | -0.23 (-0.42 , -0.05) |
|  | GA | 0.75 (0.29 , 1.96) | 157.41 (118.27 , 196.55) | 1.71 (1.51 , 1.91) | -0.14 (-0.32 , 0.05) |
|  | GG | 0.42 (0.07 , 2.62) | 214.41 (170.62 , 258.19) | 1.61 (1.31 , 1.92) | -0.17 (-0.42 , 0.09) |
| Smoking | Current Smoking |  |  |  |  |
|  | AA | reference | 156.05 (130.51 , 181.60) | 1.19 (1.01 , 1.38) | -0.60 (-0.81 , -0.39) |
|  | GA | 1.82 (0.53 , 6.30) | 141.12 (113.96 , 168.27) | 1.25 (1.02 , 1.49) | -0.55 (-0.78 , -0.32) |
|  | GG | NA | 180.51 (131.26 , 229.76) | 1.30 (1.01 , 1.60) | -0.74 (-1.02 , -0.47) |
|  | Non-smoking |  |  |  |  |
|  | AA | reference | 150.24 (137.68 , 162.81) | 1.76 (1.65 , 1.87) | -0.13 (-0.22 , -0.05) |
|  | GA | 0.79 (0.46 , 1.36) | 138.90 (17.12 , 160.68) | 1.78 (1.69 , 1.88) | -0.09 (-0.18 , -0.01) |
|  | GG | 0.96 (0.37 , 2.54) | 157.16 (129.45 , 184.87) | 1.77 (1.62 , 1.93) | -0.16 (-0.32 , -0.01) |
| Alcohol consumption | Drinker |  |  |  |  |
|  | AA | reference | 144.59 (125.92 , 163.27) | 1.72 (1.55 , 1.88) | -0.05 (-0.22 , 0.12) |
|  | GA | 0.93 (0.40 , 2.15) | 136.96 (104.04 , 169.92) | 1.78 (1.59 , 1.96) | -0.02 (-0.22 , -0.17) |
|  | GG | 0.80 (0.15 , 4.29) | 144.13 (98.34 , 189.92) | 1.82 (1.60 , 2.04) | 0.23 (-0.01 , 0.47) |
|  | Non-drinker |  |  |  |  |
|  | AA | reference | 157.04 (129.33 , 184.76) | 1.60 (1.43 , 1.77) | -0.22 (-0.34 , -0.09) |
|  | GA | 0.88 (0.47 , 1.63) | 150.84 (117.19 , 184.50) | 1.65 (0.49 , 1.80) | -0.16 (-0.28 , -0.04) |
|  | GG | 0.95 (0.30 , 3.04) | 182.22 (147.18 , 217.27) | 1.57 (1.36 , 1.79) | -0.26 (-0.43 , -0.08) |
|  |  |  |  |  |  |
|  |  |  |  |  |  |
|  |  |  |  |  |  |
| rs7331124 |  |  |  |  |  |
| Physical activity | Sedentary |  |  |  |  |
|  | CC | reference | 143.78 (123.45 , 164.11) | 1.73 (1.60 , 1.87) | -0.11 (-0.21 , -0.01) |
|  | TC | 1.43 (0.61 , 3.36) | 141.40 (111.90 , 170.90) | 1.71 (1.54 , 1.88) | -0.12 (-0.26 , 0.01) |
|  | TT | NA | NA | NA | NA |
|  | Non-sedentary |  |  |  |  |
|  | CC | reference | 160.02 (131.44 , 188.59) | 1.63 (1.44 , 1.82) | -0.20 (-0.37 , -0.03) |
|  | TC | 0.64 (0.12 , 3.46) | 149.37 (101.47 , 197.26) | 1.73 (1.47 , 1.98) | -0.10 (-0.32 , 0.12) |
|  | TT | NA | NA | NA | NA |
| Smoking | Current Smoking |  |  |  |  |
|  | CC | reference | 161.61 (143.37 , 179.85) | 1.22 (1.05 , 1.40 ) | -0.58 (-0.75 , -0.40) |
|  | TC | 2.42 (0.37 , 15.71) | 118.23 ( 83.30 , 153.16) | 1.27 (0.98 , 1.57) | -0.47 (-0.75 , -0.19) |
|  | TT | NA | NA | NA | NA |
|  | Non-smoking |  |  |  |  |
|  | CC | reference | 148.54 (137.54 , 179.54) | 1.77 (1.68 , 1.86) | -0.12 (-0.20 , -0.04) |
|  | TC | 0.88 (0.37 , 2.07) | 157.42 (133.41 , 181.42) | 1.77 (1.64 , 1.91) | -0.12 (-0.23 , -0.01) |
|  | TT | NA | NA | NA | NA |
| Alcohol consumption | Drinker |  |  |  |  |
|  | CC | reference | 140.62 (121.73 , 159.51) | 1.71 (1.56 , 1.87) | -0.08 (-0.25 , 0.09) |
|  | TC | 1.85 (0.41 , 8.31) | 147.37 (133.41 , 181.42) | 2.07 (1.82 , 2.32) | 0.23 (-0.01 , 0.47) |
|  | TT | NA | NA | NA | NA |
|  | Non-drinker |  |  |  |  |
|  | CC | reference | 157.64 (131.81 , 184.48) | 1.62 (1.46 , 1.78) | -0.19 (-0.31 , -0.07) |
|  | TC | 1.03 (0.45 , 2.38) | 155.68 (117.10 , 194.27) | 1.57 (1.39 , 1.76) | -0.23 (-0.37 , -0.08) |
|  | TT | NA | NA | NA | NA |
|  |  |  |  |  |  |
|  |  |  |  |  |  |
|  |  |  |  |  |  |
| rs8191973 |  |  |  |  |  |
| Physical activity | Sedentary |  |  |  |  |
|  | GG | reference | 155.28 (131.46 , 179.11) | 1.72 (1.58 , 1.86) | -0.12 (-0.22 , -0.03) |
|  | GC | 1.35 (0.76 , 2.39) | 126.57 (104.38 , 148.77) | 1.75 1.61 , 1.89) | -0.08 (-0.19 , 0.04) |
|  | CC | 1.12 (0.16 , 7.73) | 137.76 ( 74.86 , 200.66) | 1.73 (1.39 , 2.07) | -0.23 (-0.57 , 0.10) |
|  | Non-sedentary |  |  |  |  |
|  | GG | reference | 155.40 (128.79 , 182.10) | 1.66 (1.46 , 1.85) | -0.18 (-0.36 , 0.01) |
|  | GC | 0.92 (0.33 , 2.61) | 171.09 (127.48 , 214.69) | 1.58 (1.37 , 1.79) | -0.22 (-0.41 , -0.04) |
|  | CC | 1.76 (0.39 , 7.93) | NA | 1.70 (1.40 , 2.01) | -0.40 (-0.37 , 0.10) |
| Smoking | Current Smoking |  |  |  |  |
|  | GG | reference | 163.09 (138.56 , 187.63) | 1.23 (1.05 , 1.41) | -0.62 (-0.82 , -0.43) |
|  | GC | 0.69 (0.09 , 4.99) | 126.71 ( 95.72 , 157.69) | 1.21 (0.95 , 1.48) | -0.49 (-0.74 , -0.25) |
|  | CC | 6.79 (0.66 , 69.31) | NA | 1.16 (0.74 , 1.59) | -0.68 (-1.04 , -0.32) |
|  | Non-smoking |  |  |  |  |
|  | GG | reference | 151.49 (139.08 , 163.90) | 1.77 (1.67 , 1.87) | -0.12 (-0.20 , -0.04) |
|  | GC | 1.22 (0.69 , 2.16) | 143.20 (126.98 , 165.43) | 1.76 (1.66 , 1.86) | -0.12 (-0.21 , -0.03) |
|  | CC | 1.25 (0.32 ,4.93) | 134.00 ( 79.83 , 188.17) | 1.83 (1.59 , 2.07) | -0.12 (-0.36 ,0.11) |
| Alcohol consumption | Drinker |  |  |  |  |
|  | GG | reference | 156.38 (133.76 , 178.99) | 1.78 (1.62 , 1.94) | -0.03 (-0.20 , 0.14) |
|  | GC | 1.20 (0.47 , 3.06) | 112.17 (78.84 , 145.51) | 1.65 (1.45 , 1.85) | -0.08 (-0.29 , 0.13) |
|  | CC | 2.23 (0.30 , 16.62) | 95.01 (38.95 , 151.07) | 1.71 (1.36 , 2.06) | -0.17 (-0.62 , 0.28) |
|  | Non-drinker |  |  |  |  |
|  | GG | reference | 158.86 (132.05 , 185.67) | 1.60 (1.44 , 1.77) | -0.21 (-0.32 , -0.09) |
|  | GC | 1.11 (0.60 , 2.08) | 148.27 (110.33 , 186.20) | 1.64 (1.48 , 1.80) | -0.17 (-0.29 , -0.04) |
|  | CC | 1.57 (0.42 , 5.91) | 178.13 (140.01 , 216.26) | 1.69 (1.42 , 1.95) | -0.21 (-0.45 , -0.02) |

^1^Model adjusted for age, gender, site, ethnic population, physical activity, smoking, and alcohol consumption. ^2^Model adjusted for age, gender, region, BMI, ethnic population, physical activity, smoking, and alcohol consumption. ^3^Analysis with log transformation. ^4^No data in the category
