# Supplementary material for: Effect of Common Genetic Variants of Growth Arrest-Specific 6 Gene on Insulin Resistance, Obesity and Type 2 Diabetes in an Asian Population
Source: PLoS One. 2015 Aug 18;10(8):e0135681. doi: 10.1371/journal.pone.0135681 (PMC4540485; doi:10.1371/journal.pone.0135681)
Supplement: S5 Table — (DOCX) [file pone.0135681.s005.docx]

**S5 Table.**

| SNP | Model | obesity^1^ | SSPG^2^ | FPI^2,3^ | HOMA-IR^2,3^ |
| --- | --- | --- | --- | --- | --- |
| rs8191974 | Dominant | 0.393 | 0.065 | 0.872 | 0.573 |
|  | Recessive | 0.518 | 0.183 | 0.289 | 0.389 |
| rs7323932 | Dominant | 0.025 | 0.005 | 0.583 | 0.962 |
|  | Recessive | 0.758 | 0.907 | 0.683 | 0.604 |
| rs7331124 | Dominant | 0.444 | 0.960 | 0.093 | 0.445 |
|  | Recessive | 0.181 | 0.429 | 0.238 | 0.176 |
| rs8191973 | Dominant | 0.554 | 0.022 | 0.570 | 0.661 |
|  | Recessive | 0.772 | 0.067 | 0.947 | 0.856 |

SSPG: steady state plasma glucose, FPI: fasting plasma insulin, HOMA-IR: homeostasis model assessment of insulin resistance.

^1^Model adjusted for age, gender, site, and ethnic population.

^2^Model adjusted for age, gender, site, BMI, and ethnic population.

^3^analysis with log transformation
